# Chestnut (*Castanea sativa* Miller.) Burs Extracts and Functional Compounds: UHPLC-UV-HRMS Profiling, Antioxidant Activity, and Inhibitory Effects on Phytopathogenic Fungi

**DOI:** 10.3390/molecules24020302

**Published:** 2019-01-15

**Authors:** Tiziana Esposito, Rita Celano, Catello Pane, Anna Lisa Piccinelli, Francesca Sansone, Patrizia Picerno, Massimo Zaccardelli, Rita Patrizia Aquino, Teresa Mencherini

**Affiliations:** 1 Department of Pharmacy, University of Salerno, Via Giovanni Paolo II, 132, 84084 Fisciano (SA), Italy; tesposito@unisa.it (T.E.); rcelano@unisa.it (R.C.); apiccinelli@unisa.it (A.L.P.); fsansone@unisa.it (F.S.); ppicerno@unisa.it (P.P.); aquinorp@unisa.it (R.P.A.); 2 Ph.D. Program in Drug Discovery and Development, University of Salerno, Via Giovanni Paolo II, 132, 84084 Fisciano (SA), Italy; 3 Consiglio per la Ricerca in Agricoltura e l’Analisi dell’Economia Agraria, Centro di Ricerca Orticoltura e Florovivaismo, via Cavalleggeri 25, I-84098 Pontecagnano Faiano (SA), Italy; catello.pane@crea.gov.it (C.P.); massimo.zaccardelli@crea.gov.it (M.Z.)

**Keywords:** chestnut burs, hydrolysable tannins (HTs), flavonols, antiradical and antifungal activity, *Alternaria alternata*, *Fusarium solani*, *Botrytis cinerea*

## Abstract

Chestnut (*Castanea sativa* Miller.) burs (CSB) represent a solid waste produced during the edible fruit harvesting. Their usual disposal in the field increases the environmental and economic impact of the agricultural process. HPLC-UV-HRMS profiling revealed that CSB organic and aqueous extracts (CSB-M, CSB-H, CSB-A) contain several hydrolyzable tannins, mainly ellagitannins, and glycoside flavonols. Ellagic acid (EA) and chestanin are predominant components (5–79 and 1–13 mg/g dry extract, respectively). NMR analysis confirmed the chemical structures of the major constituents from CSB-M. The extracts displayed a significant scavenging activity against DPPH^•^ (EC_50_ 12.64–24.94 µg/mL) and ABTS^•+^ radicals (TEAC value 2.71–3.52 mM Trolox/mg extract). They were effective in inhibiting the mycelial growth (EC_50_ 6.04–15.51 mg/mL) and spore germination (EC_50_ 2.22–11.17 mg/mL) of *Alternaria alternata* and *Fusarium solani*. At the highest concentration, CSB-M was also active against *Botrytis cinerea* both in mycelium and spore form (EC_50_ 64.98 and 16.33 mg/mL). The EA contributed to the antifungal activity of extracts (EC_50_ on spore germination 13.33–112.64 µg/mL). Our results can support the upgrading of chestnut burs from agricultural wastes to a resource of natural fungicides for managing fruit and vegetable diseases.

## 1. Introduction

Italy, with France, Spain, and Portugal, is one of the largest European producers of sweet chestnuts (*Castanea sativa* Mill., Fagaceae family), and Campania region covers about 40% of the national fruit crop [1]. The harvest takes place manually after the fall of the fruits, usually starting from the beginning of September, for the more precocious varieties, until the beginning of November for the later ones. Chestnuts represent a nutritional complement in human diet for the high content in starch, carbohydrates, and unsaturated (omega-3) fatty acids [2,3]. The unprocessed fruit, consumed as it is, represents half of the production. Its transformation in gluten-free flour and *marron-glacé* represents the remaining part [1]. The eatable part is the 85% *w*/*w* of the whole nut; the harvest and processing phases produce large amounts of by-products and wastes, such as leaves, burs, outer and inner teguments. Currently, it is possible to use as industrial fuel only the inner teguments, released during the peeling phase of chestnuts [4]. However, different *C. sativa* by-products have potential properties for application in pharmaceutical, cosmetic, food and leather industry, because of occurring specific compounds [5,6]. Burs represent about 20% *w*/*w* of the total chestnut weight and usually remain in the field after the harvesting, favoring the proliferation of insect larvae. The strategy of farmers to avoid crop damages is to burn the wastes [1]. The strategy of researches is to exploit and reuse these solid residues, aiming to reduce both the environmental and economic impacts of the agro-industrial process also getting new products. Burs have a low protein and lipid content, but a high concentration (60–80%) of indigestible carbohydrates (lignans, pectins, dietary fibers). Chestnut burs also present essential (arginine and leucine, from 749 to 205 mg/100 g) and non-essential (aspartic and glutamic acid, and proline, from 461 and 190 mg/100 g) aminoacids. The lipid extract contains tocopherols (α-, β-, γ- and δ-) and tocotrienols (α-, β-, γ- and δ-) in concentration from 318 to 3.04 mg/100 g [7]. Glucose esters with gallic acid (-mono, -di, and -trigalloylglucose), ellagitannins (vescalagin/castalagin), phenolic acids (gallic, ellagic, protocatechuic, cholorgenic acid), flavonoids (apigenin, quercetin, and quercetin 3-*O*-β-glucoside) has been identified in chestnut burs [4,6,8]. Nevertheless, information about chestnut burs composition is still lacking, and a comprehensive chemical investigation of secondary metabolites has never been carried out. According to their significant total phenol content, the chestnut bur extracts displayed marked antioxidant properties. Polyphenols act as antioxidants (AH) giving hydrogen atoms from their hydroxyl groups to lipid radicals (R•/RO•/ROO•) [9]. The reaction (Reaction 1) produces lipid derivatives and phenolic radicals. The delocalization around the aromatic ring of unpaired electron stabilizes the oxidated polyphenols (A•). The antioxidant radical (A•) can take part in the termination reactions producing non-radical molecules (Reaction 2):

R•/RO•/ROO• + AH → A• + RH/ROH/ROOH(1)

RO•/ROO• + A• → ROA/ROOA(2)

Thus, polyphenols potentially act as natural protective agents both against human oxidative stress-mediated pathologies [7], and lipid oxidation and microbial spoilage in foods [9]. Polyphenols and polyphenol-rich extracts show a broad-spectrum of biological, including antimicrobial, efficacy, both against human pathogens [10,11,12] and bacterial and fungal strains causing plant infections [13,14,15,16,17]. Phytopathogenic fungi are damaging for fruit and vegetable productive chain, causing both in field and post-harvest yield losses and food decay also finding out serious risks for consumers, because of dangerous secondary metabolites production like mycotoxins [16,17]. The fungi *Alternaria alternata*, *Botrytris cinerea*, and *Fusarium solani* are among the most common pathogens blame for mold and rot in many crops. A limited number of authorized synthetic fungicides acts in their management. Their widespread use involves developing resistant strains and raises environmental and human health concerns because of persistent chemical residues [16]. To overcome these major drawbacks, an attractive alternative or a complementary mean to synthetic antimicrobial is getting natural derivatives from plants or agro-industrial residues, having a nontoxic and biodegradable profile. Glazer et al. [16] proved the ability of an aqueous *Punica granatum* peel extract, rich in polyphenols and hydrolysable tannins (mainly ellagitannins), to reduce the mycelial growth of *A. alternata* and *F. solani* at concentrations of 8.60 and 17.20 mg/mL, respectively. The positive correlation between the in vitro and in vivo antifungal activity of *Capsicum annuum* extracts (between 5–25 mg/mL) against *A. alternata* on cherry tomato fruits [15] was associated to their phenolic (gallic acid, caffeic acid, catechin) content. In literature there are no scientific research reporting the antifungal activity of chestnut bur extracts against phytopathogenic fungi. However, the efficacy of an aqueous bur extract against *S. aureus* with a MIC value of 5 mg/mL, and the ineffectiveness against *P. aeruginosa*, *E. coli* and *C. albicans* up to 50 mg/mL was demonstrated [18]. A bark chestnut-extracted colorant showed a wide spectrum of antifungal activity against seed-borne pathogens inhibiting the mycelial growth of *Alternaria dauci*, *Alternaria radicina*, *Colletotrichum lindemuthianum*, and *Ascochyta rabiei* [19]. The potential of *C. sativa* burs (CSB) as source of phenolic compounds with antioxidant and antimicrobial activity could be evaluated. CSB is a poorly explored solid waste and very limited data are available on its chemical characterization. In this study, the ultra-high-performance liquid chromatography coupled with UV and high-resolution mass spectrometry detectors (UHPLC-UV-HRMS) provided an accurate profiling of CSB organic and aqueous extracts. For the first time, the quantification of chestanin in chestnut by-products, and the ellagic acid levels in chestnut burs were carried out. 1D- and 2D-NMR experiments confirmed the chemical structures of the major constituents making possible the determination of their amount in CSB extracts. The in vitro scavenging activity against the radicals DPPH^•^ and ABTS^•+^ was verified. CSB extracts and marker compounds were also evaluated as inhibitive effect on mycelial growth and spore germination of *Alternaria alternata*, *Fusarium solani*, and *Botrytis cinerea*.

## 2. Results and Discussion

### 2.1. Total Phenol Content and Antioxidant Activity of CSB Extracts

Chestnut burs, recovered during the harvesting of edible fruit, were powdered and extracted with three different procedures in order to define the influence of solvent and method on the total polyphenol content, and efficacy against free radicals and phytopathogenic fungi. Fernández-agulló et al. [4] compared the use of different solvent systems (aqueous methanol and ethanol, and water) for the recovery of antioxidants from chestnut burs, obtaining extraction yield in the ranges of 12.91–19.58, 11.13–18.38 % (*w*/*w*) from aqueous methanol and ethanol, and 8.54–17.35 % (*w*/*w*) from water, respectively. The highest Total Phenolic Content (TPC) was registered for the aqueous methanol (50%, *v*/*v*) extract (17.74–27.69 g GAE/100 g extract) obtained at 75 °C. In our study, an exhaustive maceration at room temperature using solvents with increasing polarity, to obtain the extract CSB-M, was compared to extraction procedures by aqueous ethanol under stirring at 45 °C, and boiling water that allowed to collect the extracts CSB-H and CSB-A, respectively. The selection of ethanol and water is based on their classification as GRAS (Generally Recognized as Safe) solvents, with the advantage of eco-sustainability and potential application in the food industry. In our conditions, the use of aqueous ethanol (50%, *v*/*v*) led to the highest extraction yield (11.6% *w*/*w*), with a value close to the range of the above-mentioned work (11.13–18.38%) [4]. However, the sequential maceration with hexane, chloroform, and methanol (CSB-M extract) represented the most efficient method to recover polyphenols within the alcoholic phase, confirming previous data that we have obtained with other food by-products, such as the hazelnut skins [20]. In fact, as shown by the Folin-Ciocalteu results (Table 1), CSB-M possessed a significantly (*p* < 0.05) higher TPC (26.42 g GAE/100 g extract) than CSB-H and CSB-A (20.60 and 20.26 g GAE/100 g extract, respectively). The determination of Pearson correlation coefficients (r) defined the linear relationship between TPC and extracts scavenging activity against DPPH^•^ and ABTS^•+^ radicals (r = −0.9422 and 0.836, respectively). The comparable (*p* > 0.05) activity of CSB-H and CSB-A as scavengers of both radicals (EC_50_ 24.94 and 22.38 µg/mL, TEAC value 3.00 and 2.71 mM Trolox/mg extract, respectively) (Table 1) was derived from the similar TPC (20.60 and 20.26 GAE g/100g extract). On the other hand, CSB-M, which is richer in functional compounds (26.42 g GAE/100 g extract), was also the most effective against free-radicals (EC_50_ 12.64 µg/mL, TEAC value = 3.52 mM Trolox/mg extract). Quercetin 3-*O*-β-d-glucopyranoside (**25**) and ellagic acid (EA, **27**), are components of CSB extracts (see Section 2.2.), and were confirmed as strong antiradical compounds. On the contrary, chestanin (**21**) showed weak efficacy against DPPH^•^ (EC_50_ 16.62 µg/mL) and ABTS^•+^ (TEAC value 1.05 mM Trolox/mM compound) (Table 1).

### 2.2. UHPLC-UV-(−)-HRMS Profiling of CSB Extracts

**Figure 1:**
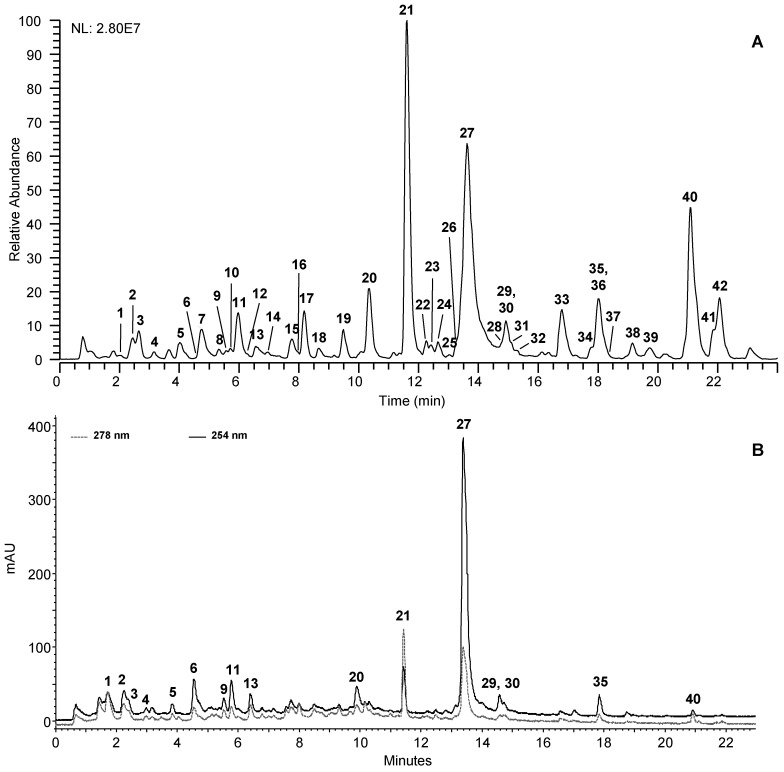
UHPLC-(−)HRMS (**A**) and UHPLC-UV (**B**) profiles of CSB-M.

CSB extracts were analyzed by UHPLC-UV-HRMS^n^ to investigate their qualitative phenolic profiles. Analyses were performed in negative ion mode due to the higher sensibility of detection for the most of detected CSB compounds. CSB-M extract disclosed richer and more complex composition than CSB-H and CSB-A; Figure 1 shows its HRMS and UV profile. Metabolite assignments were made comparing retention time and MS data of detected compounds with standard compounds, whenever available, or interpreting MS data (accurate masses and MS/MS fragment ions) combined with chemo-taxonomic data reported in the literature and databases. UHPLC-UV-HRMS^n^ profiling allowed to identify 42 compounds in CSB that can be grouped into two major classes of secondary metabolites: hydrolysable tannins (HTs) and flavonoids (Table 2).

#### 2.2.1. Hydrolysable Tannins

HTs represent a large group of polyphenolic compounds and they were one of the main class of compounds reported in chestnut by-products [6,21]. HTs can be divided in simple gallic acid derivatives, gallotannins, and ellagitannins [22]. Gallic acid (GA) represent the building block of a wide variety of HTs, from simple monomers to multiple oligomers [23]. EA (**27**), identified by comparison with pure compound, was the main compound of CSB extracts. It is the product of spontaneous intramolecular dilactonization of hexahydroxydiphenoyl (HHDP) acid. EA may exists in plant materials as free compound or may be generated by hydrolysis of ellagitannins containing HHDP group. In addition to EA, two its trimethyl glycoconjugates (**28**, trimethyl-ellagic acid hexoside, and **36**, trimethyl-ellagic acid deoxyhexoside) were also identified as constituents of CSB (Table 2). HRMS/MS spectra of both compounds were dominated by the product ion at *m*/*z* 343.0449 due to the removal of sugar moieties (hexose and deoxyhexose, respectively). Along with EA (**27**), chestanin (**21**) was identified as the most abundant component of CSB extracts. Other four analogous compounds, characteristic of chestnut, were detected in CSB extracts: chesnatin (**17**), isochesnatin (**19**), cretanin (**20**) and chestanin isomer (**24**). Molecular formulas of **21** and **20**, C_40_H_42_O_26_ and C_20_H_22_O_13_, suggested that **21** derived by oxidative coupling process of two molecules of **20**. Product ion at *m/z* 467.0817 ([M – H − C_20_H_22_O_13_]^–^) in HRMS/MS spectrum of **21**, generated by the loss of one **20** molecule, supported further the relation between **21** and **20**. Additionally, HRMS/MS spectra of **20** and **21** showed [M – H − C_13_H_16_O_8_]^–^ ions at *m*/*z* 169.0139 and 637.1036, corresponding to elimination of glucosyl 3,4,5-trihydroxybenzyl alcohol unit (Table 2). Based on this evidence, **20** and **21** were identified as cretanin and chestanin, respectively. Their structures were subsequently confirmed by NMR analysis (see Section 2.3). HRMS data of compound **24** matched up to those of chestanin (**21**), so it was recognized as its isomer (Table 2). Molecular formulas of the isomers **17** and **19** (C_27_H_26_O_18_) indicated the occurrence of one more galloyl unit than cretanin (**20**). These isomers were differentiated by HRMS/MS spectra, that resulted different regarding the loss of terminal GA unit. Compound **17** displayed an abundant ion at *m*/*z* 467.0813 produced by the elimination of GA molecule, while **19** presented two main product ions at *m*/*z* 593.1131 and 469.0973 related to the loss of terminal GA unit. These MS^2^ data provided evidence that **17** and **19** differ in the C–O diaryl ether bond, and the structures of chesnatin and isochesnatin were proposed for **17** and **19**, respectively, then confirmed by NMR (see Section 2.3.). The ellagitannins (ETs) identified in the CSB extracts, resulted to be the most abundant class of HTs. ETs derived from gallotannins by oxidative coupling of adjacent galloyl groups [24]. The basic structure for most ETs is HHDP group, which derives from oxidative C–C coupling between two spatially adjacent galloyl groups of n-galloylglucose [24]. HHDP group by coupling to a third galloyl group, can form the nonahydroxytriphenoyl (NHTP) group, characteristic of *C*-glycosidic ETs with an open glucose core. Alternatively, HHDP group can undergo a double oxidation and generate the chebuloyl group [24]. Molecular formulas of ETs calculated by accurate masses (Table 2) allowed to establish the number and type of groups (galloyl, HHDP, NHTP) linked to the glucose moiety. According to the approach proposed by Moilainen et al. [24], the molecular formulas of ETs containing HHDP or NHTP group differ by –2H or –4H, respectively, than the related galloyl-glucoses. As an example, the molecular formula of compound **2** (C_41_H_26_O_26_, –6H than pentagalloyl-glucose C_41_H_32_O_26_), suggested the structure of HHDP-NHTP-glucose. In the case of compound **12** (C_41_H_28_O_26_, –4H than pentagalloyl-glucose C_41_H_32_O_26_), two structures may be proposed: galloyl-diHHDP-glucose or digalloyl-NHTP-glucose. In this case, HRMS/MS spectra provided important information to elucidate ET structures. Generally, ETs containing HHDP display the characteristic product ion [M − H − EA]^−^ due to the elimination of HHDP group as EA. Likewise, ETs with galloyl group on glucose moiety, present [M − H − galloyl]^−^ ions in MS/MS spectra. Moreover, the losses of H_2_O and CO_2_ from [M − H]^−^ are characteristic of C-glycosidic ETs and of a free carboxyl group, respectively [24]. Thus, product ions at *m*/*z* 467.0813 ([M − H − EA]^–^) and 783.0690 ([M − H − galloyl]^–^) allowed to assign the structure of galloyl-diHHDP-glucose to **12**. Using this approach and the informations from HPLC-HRMS and MS/MS (Table 2), four ETs containing exclusively HHDP group (**5**, **8**, **12** and **15**) were tentatively identified in CSB: one diHHDP-glucose (**5**, pedunculagin), one galloyl-diHHDP-glucose (**12**, stachyurin or casuarinin) and two digalloyl-HHDP-glucose (**8** and **15**, tellimagrandin I). In addition, two ETs with a NHTP group, **2** (castalagin or vescalagin) and **4** (methylvescalagin), were detected as constituents of CSB-M extract. Molecular formulas (C_41_H_30_O_27_) of the isomers **6** and **16** contain one additional water molecule than **12** (C_41_H_28_O_26_), suggesting the presence of a chebuloyl group generated by oxidation of HHDP moiety [24]. This was further supported by the characteristic product ions at *m/z* 909.0974 and 785.0809, corresponding to the loss of carboxylic ([M − H − CO_2_]^–^) and chebuloyl group ([M − H − C_7_H_4_O_5_]^–^), respectively, in MS/MS spectra of **6** and **16**. Based on these evidence, the structure of chebulagic acid was tentatively assigned to isomers **6** and **16**. Two methyl esters of chebulagic acid, **14** and **18**, were also detected in CSB. These differed from chebulagic acid isomers **6** and **16** by one methyl group (C_42_H_32_O_27_) and presented the elimination of methylchebuloyl group ([M − H − C_8_H_6_O_5_]^–^) from [M − H]^–^ ion (Table 2). In ETs, galloyl groups may be attached to HHDP group also via ether bonds (C–O–C), as in the valoneoyl group [24]. In this case, the molecular weight of an ET increase by 168 Da (+C_7_H_4_O_5_), so the ether type galloylation may be distinguished on the basis of molecular formula of ET. Again, the valoneoyl group, that presents a free carboxylic acid, is detected by the characteristic product ion [M − H − CO_2_]^–^ in MS/MS spectra [24]. Two ETs with valoneoyl group (**1** and **10**) were detected in CSB and they were tentatively identified as castavaloninic or vescavaloninic acid (Table 2). In CSB also one dimeric ET (**13**, C_82_H_52_O_52_) was identified. It was detected as double charged molecular ion [M − 2H]^2–^ at *m*/*z* 933.0609, and MS fragmentation provided important information about the structure of two ET monomer tentatively identified as HHDP-NHTP-glucose (**2**) and galloyl-diHHDP-glucose (**12**) according to the typical product ions of these two ETs in the MS/MS spectra of **13** (*m*/*z* 915 and 631 for **2** and *m*/*z* 633 for **12** (Table 2). Based on these evidences the structure of cocciferin d2 was assigned to compound **13**.

Finally, three ET metabolites (**7**, **9** and **11**) were detected in CSB. These compounds were identified as castacrenin A-C isomers (C_27_H_18_O_17_). They showed different relative intensities of the product ions [M − H − C_3_H_6_O_3_]^–^ and [M − H − C_4_H_8_O_4_]^–^ due to the cleavage of glycosidic chain (Table 2). The isolation procedure and NMR analysis allowed to differentiate the three isomers.

#### 2.2.2. Flavonoids

Flavonoids constitute the second representative class of CSB secondary metabolites, according to occurrence data on chestnut by-products [6,21]. All detected flavonoids were flavonol glycoconjugates, particularly, quercetin (**23**, **25**, **26**, and **33**), isorhamnetin (**30**–**32**, **37**) and kaempferol derivatives (**29**, **34**, **35**, **38**–**42**). Different sugars (mainly hexose, deoxyhexose, hexuronose), often acylated with aliphatic (acetyl) or aromatic (galloyl, coumaroyl, and caffeoyl) groups were linked to flavonol aglycones (Table 2). The proposed CSB flavonol structures were confirmed by comparison with pure compounds (isolated or standards), when available, or identified on the basis of the accurate masses of precursor and product ions, the fragmentation pattern of the aglycone (MS^3^ experiments), literature data and following the spectra interpretation guidelines for flavonoids [25,26,27]. To date, this study represents the first report on the profiling of phenolic compounds in chestnut burs. Among the identified compounds, little research in literature have previously reported only the occurrence of EA, vescalagin/castalagin, and quercetin 3-*O*-β-glucoside in chestnut burs [8]. In addition, the present research provides an accurate characterization of most HTs by HRMS/MS. The lack of available literature data makes our results a valuable contribution to the elucidation of this metabolite class by MS techniques.

### 2.3. Isolation and Identification of Compounds

**Figure 2:**
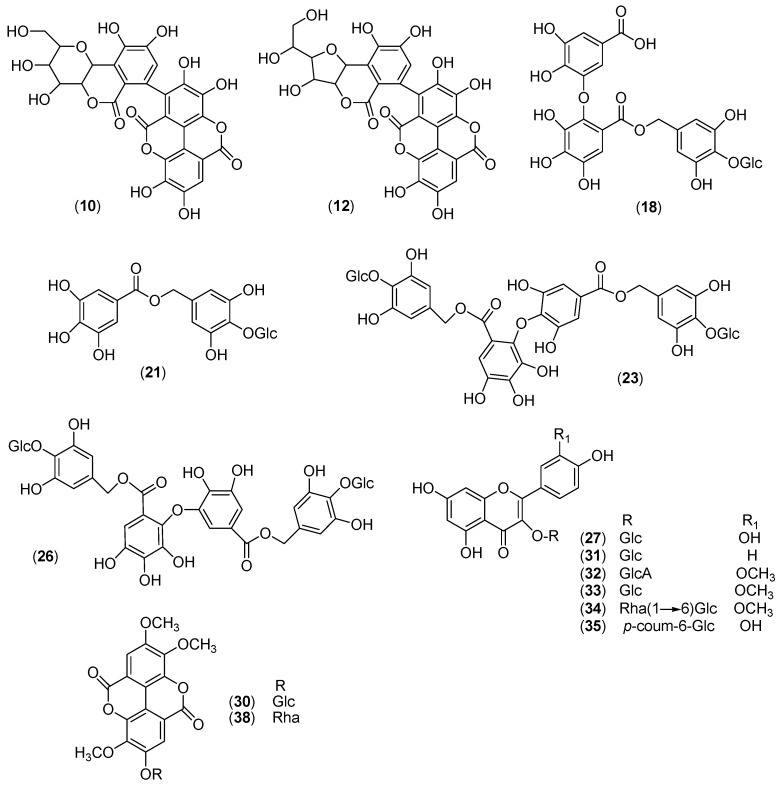
Secondary metabolites isolated from CSB-M extract. Glc = β-d-glucopyronoside; Rha = α-l-rhamnopyranoside; GlcA = β-d-glucuronopyranoside; *p*-coum = -para-coumaroyl.

In order to isolate the main CSB constituents, CSB-M was partitioned between *n*-BuOH and H_2_O, and the organic portion was subjected to fractionation, using gel permeation and semipreparative RP-HPLC chromatographic columns. The chemical structures of purified compounds were elucidated by spectroscopic data in 1D- and 2D- NMR experiments (Figure 2). The procedure allowed to confirm the structures proposed by HRMS analysis of the GA (chesnatin **17**, isochesnatin **19**, cretanin **20** and chestanin **21**), and EA derivatives (**28** and **36**), and to differentiate three ET metabolite isomers (castacrenin A-C **7**, **9** and **11**). Regarding flavonoids, kaempferol and isorhamnetin glycosides (**29**, **30**, and **32**) were also confirmed. Moreover, the structure of flavonols **25**, **31** and **33** were unambiguously assigned to quercetin 3-*O*-β-d-glucopyranoside, isorhamnetin 3-*O*-β-d-glucopyranoside, and quercetin 3-O-(6″-​*O*-​trans-​*p*-​coumaroyl)​-​β-​d-​glucopyranoside, respectively, studying the proton coupling constants, 1D-TOCSY, ^1^H-^1^H DQF-COSY, ^1^H-^13^C HSQC, and HMBC experiments of the glycosidic units. The isolation procedure also led to obtain pure chestanin (**21**), not commercially available, used as standard compound in the quantitative analysis of CSB extracts.

### 2.4. Quantitative Analysis of CSB Extracts

HPLC profiling of CSB extracts revealed that chestanin (**21**) and EA (**27**) were the most abundant compounds of chestnut burs (Table 1). According to literature data, both compounds were detected in chestnut bark and by-products [4,6]. No significant differences were observed in the qualitative profiles of three different CSB extracts (CSB-M, A, and H). Thus, chestanin and EA amounts represent useful quantitative markers for the characterization of CSB extracts. To advance understanding of CSB applications, a quantitative evaluation of its main compounds in three different extracts (CSB-M, CSB-A, and CSB-H) was performed. Chestanin (**21**) and EA (**27**) contents were estimated by UHPLC-UV analysis using external calibration method, and the data are listed in Table 1. Chestanin and EA amounts varied according to the applied extraction conditions. CSB-M extract presented the highest content of both analyzed compounds (79.32 mg g^−1^ of chestanin and 13.34 mg g^−1^ of EA) compared to CSB-A and CSB-H extracts. The latter extracts showed instead comparable levels of both compounds. Data on the levels of phenolic constituents in chestnut bur are absent in literature. Recently, Squillaci et al. [34] reported an EA amount of 0.6–0.8 mg/g dry extract in inner and outer chestnut shells. In this regard, to date our study is the first work reporting the quantification of chestanin in chestnut by-products and the EA levels in chestnut burs.

### 2.5. In Vitro Antifungal Activity

The control of *Alternaria alternata*, *Botrytis cinerea*, and *Fusarium solani* infections is a challenge for cropping, storage and commercial phases of vegetable foods, due to the frequent resistance developed by pathogens against the applied synthetic molecules, regulatory limitations in the use of fungicides, and public concerns about healthy and eco-friendly foods. EA, one of the most active polyphenols in CSB extracts, has good antioxidant properties and proved to be suitable for postharvest kumquat treatments to preserve fruit quality [12,35]. In order to evaluate the potential use of CSB extracts for the management of field and or postharvest vegetable diseases, the inhibitory effects of CSB-M, CSB-H, and CSB-A on mycelial growth and spore germination of the selected fungi were studied by using an amended-plate technique and a liquid microculture method, respectively [15]. *A. alternata* and *F. solani* resulted as the most sensitive pathogens, with EC_50_ of all extracts varying from 6.04 mg/mL to 15.51 mg/mL (Table 3), while *B. cinerea* resulted in the less sensitive pathogen (Table 3). Among the three extracts, CSB-M was the most active against the phytopathogens showing the lowest EC_50_ values (Table 3) against *A. alternata,* and *F. solani* (6.29 mg/mL and 6.04 mg/mL, respectively). In addition, CSB-M completely inhibited mycelial growth of both *A. alternata,* and *F. solani* at 30 mg/mL. On the contrary, CSB-H and CSB-A were not detrimental for *B. cinerea* growth up to 70 mg/mL; the EC_50_ determined only at a high concentration of CSB-M (64.98 mg/mL, Table 3).

The inhibitory effect of CSB extracts on fungal conidia germination was also investigated. The spore cells represent the microorganism form able to reproduce and survive in adverse conditions and are usually very resistant even to the strongest treatments [36]. Our results (Table 4) showed that phytopathogenic fungi are most sensitive to CSB extracts in the phases of spore germination than those of saprophytic mycelial growth; *A. alternata*, and *F. solani* being the most sensitive strains (EC_50_ values between 2.22 and 11.17 mg/mL). CSB-M proved to be the most effective extract against *A. alternata* and *F. solani* with EC_50_ values of 2.66 mg/mL and 2.22 mg/mL, respectively (Table 4), while exerted weak effect (EC_50_ 16.33 mg/mL, Table 4) on *B. cinerea* spores.

To understand the role of the predominant CSB compounds in determining the antifungal behavior of the extracts, pure chestanin (**21**) and EA (**27**) were tested singularly on the spore germination. Their effects were compared with those of two synthetic fungicides, such as iprodione, commonly used, under regulation, for the control of postharvest diseases caused by *A. alternata* and *B. cinerea*, and carbendazim used against *F. solani* infections [37,38] (Table 5). EA (**27**) resulted the most active compound against all fungi, with EC_50_ included between 13.33–112.64 μg/mL. Instead, chestanin (**21**) was the less active, with EC_50_ value against *A. alternata* of 561.56 μg/mL, and higher than 2 mg/mL against *B. cinerea* and *F. solani*.

Different functional properties of EA are reported in literature from antioxidant to antimicrobial and anti-inflammatory activities [11,12]. The antimicrobial activity of EA was reported against different *Candida* strains, with a MIC value ranging from 25 to 100 μg/mL [11] and against phytopathogenic fungi *F. solani* and *B. cinerea* at concentrations of 1 mg/mL and 390 µg/mL, respectively [17,35]. In a previous investigation, EA showed a dual behavior against *B. cinerea*, resulting detrimental for germ tube length and mycelial growth and ineffective on spore germination at low concentration (18 ppm); whereas the higher dose of the compound incited both germination and in vitro growth (90 ppm) [14]. Here, findings could suggest that EA contributed to the highest effectiveness of CSB-M in both performed assays, even if the different comeback in antifungal effect of CSB extracts against target fungal species seem to be more related to the TPC than to a single isolated compound. Indeed, EC_50_ calculated on germination and plate growth of *A. alternata* and *F. solani* was well correlated with TPC (Pearson coefficient from −0.72 to −0.99). Plant-deriving polyphenols have been widely proposed as biofungicides or as adjuvants for enhancing activities of other antimicrobial drugs because of their recognized antifungal properties. Hypothetical mechanisms underlying the antimicrobial action of this wide class of secondary metabolites concern alteration of membrane integrity, impairment of cell wall with subsequent deformation and or lysis of hyphae and spore [39].

## 3. Experimental Section

### 3.1. Chemicals and Reagents

Analytical grade *n*-Hexane, Chloroform (CHCl_3_), methanol (MeOH) and ethanol (EtOH), dimethyl sulfoxide (DMSO), deuterated methanol (99.8%, CD_3_OD), Folin-Ciocalteu phenol reagent, 1,1-diphenyl-2-picrylhydrazyl radical (DPPH), 2,2′-azino-bis (3-ethylbenzothiazoline)-6 sulphonic acid (ABTS), Trolox, ellagic acid, carbendazim 97%, iprodione, and HPLC-grade methanol (MeOH) were purchased from Sigma-Aldrich (Milan, Lombardia, Italy). Potato Dextrose Agar (PDA) and Potato Dextrose Broth (PDB) were purchased from Thermo Fisher Diagnostics S.p.A (Milano, Italy). HPLC-grade water (18 mΩ) was prepared by a Milli-Q_50_ purification system (Millipore Corp., Bedford, MA, USA).

### 3.2. General Experimental Procedures

A Bruker DRX-600 NMR spectrometer, operating at 599.19 MHz for ^1^H and 150.858 MHz for ^13^C, using the TopSpin 3.2 software package, was used for NMR experiments in CD_3_OD. Chemical shifts are expressed in δ (parts per million) referring to the solvent peaks δ_H_ 3.31 and δ_C_ 49.05 for CD_3_OD, with coupling constants, *J*, in Hertz. Conventional pulse sequences were used for ^1^H-^1^H DQF-COSY, ^1^H-^13^C HSQC, and HMBC experiments [40]. HPLC analyses were performed on a Platin Blue UHPLC system (KNAUER GmbH, Berlin, Germany) consisting of two Ultra High-Pressure Pumps, an autosampler, a column temperature manager and a diode array detector, coupled to a LTQ Orbitrap XL (Thermo Scientific, San Jose, CA, USA) equipped with a electrospray ionization (ESI) probe. The data were acquired and processed with Xcalibur 2.7 software from Thermo Scientific. Chromatography was performed over Sephadex LH-20 (Pharmacia, Uppsala, Sweden). Thin-layer chromatography (TLC) analysis was performed with Macherey−Nagel precoated silica gel 60 F_254_ plates (Delchimica, Naples, Italy), and the spray reagent cerium sulfate (saturated solution in dilute H_2_SO_4_) and UV (254 and 366 nm) were used for the spot visualization. Semireparative HPLC separations were conducted on a Waters 590 series pumping system, equipped with a Waters R401 refractive index detector, a Rheodyne injector (100 μL loop), and Luna C_8_ (250 × 10 mm i.d., 10 µm, Phenomenex Inc., Castel Maggiore (BO), Italy) and C_18_ Synergy Fusion–RP 4 µm 80A (250 × 10.0 mm, Phenomenex Inc.) columns.

### 3.3. Chestnut Spiny Burs Material

Chestnut burs (from *Castanea sativa* Mill. specie) were collected in a chestnut plantation (Società Cooperativa Agricola “Castagne di Montella”) in Montella (AV), Italy, during the chestnut collection in the middle of October 2016. The burs were air-dried till equilibrium humidity and ground in a Grindomix (mod. RM 100, Retsch, Bergamo, Italy) 8000 rpm for 4 min.

### 3.4. Preparation of Chestnut Spiny Bur Extracts

Chestnut dried burs (1000 g) were sequentially defatted with *n*-hexane and chloroform, and extracted (at 25 °C) with methanol to give 71.7 g of residue (CSB-M). The extraction yield, gravimetrically determined (balance Denver Instruments-PK-201, max 2400 g d = 0.1 g; +15/30 °C), and expressed as the weight percentage of the dry matter compared to the total amount of the dry raw powder, was 7.2%, *w*/*w*. A portion of the methanol extract was partitioned between *n*-butanol and water to obtain a *n*-BuOH-soluble portion (CSB-B). A sample (50 g) of dried chestnut burs was extracted with 50% aqueous ethanol (2500 mL) by homogenization with an Ultra-Turrax T-25 (IKA ULTRA-TURRAX T25 digital) at 10,000 rpm for 4 min. The homogenate was transferred in an orbital shaker with temperature control (45 °C), and the shaking rate was set at 300 rpm for 30 min. The resulted extract was filtered through a sieve with 45 µm pore size. The solvent was evaporeted in a Buchi R-210 rotavapor (Buchi Italia srl, Milan, Italy) for the alcoholic portion, and by lyophilizer (Alpha 1–2 LD freeze dryer, Martin Christ, Germany) for the aqueous one to obtain a dry powder (CSB-H, extraction yield of 11.6%, *w*/*w*). An aqueous extract was prepared boiling 2 g of chestnut burs at 100°C for 10 min with 200 mL of distilled water. The mixture was left to stand at room temperature for 5 min, then filtered through cheesecloth, and freeze-dried using an Alpha 1–2 LD freeze dryer (Martin Christ, Germany) to obtain the dried CSB-A extract (yield 1.3%, *w*/*w*).

### 3.5. Quantitative Determination of Total Phenol Content

The Total Phenolic Content (TPC) of CSB-M, CSB-H and CSB-A was determined using the Folin-Ciocalteu colorimetric method [41]. The results were expressed as Gallic Acid Equivalents (GAE g/100 g of extract, means ± standard deviation of three determinations).

### 3.6. Bleaching of the Free-radical 1,1-Diphenyl-2-picrylhydrazyl (DPPH Test)

The radical scavenging activities of CSB-M, CSB-H, CSB-A, and pure compounds were assayed using the sTable 1,1-diphenyl-2-picrylhydrazyl radical (DPPH^•^), according to our previously reported procedures [20]. The DPPH^•^ solution (25 mg/mL in methanol, prepared daily) was kept to react 10 min with 37.5 µL of various concentrations of each sample under investigation in MeOH, EtOH:H_2_O 1:1, *v*/*v*, or H_2_O solutions (ranged from 0.5 to 100 μg/mL). After 10 min, the decrease in absorbance was measured at 517 nm (Thermo Evolution 201 UV-visible spectrophotometer, Thermo Fisher Scientific Italia, Milan, Italy). Gallic acid was used as positive control. EC_50_ (mean effective scavenging concentration) was determined as the concentration (in micrograms per milliliter) of sample necessary to decrease the initial DPPH^•^ concentration by 50%. All tests were performed in triplicate. A lower EC_50_ value indicates stronger antioxidant activity.

### 3.7. Trolox Equivalent Antioxidant Capacity (TEAC) Assay

TEAC assay was performed according to the method of Re et al. [42]. The radical cation ABTS^•+^ was generated by mixing (1:1, *v*/*v*) ABTS^•+^ (7.0 mM) and potassium persulfate (2.45 mM). The mixture was allowed to stand overnight at room temperature in the dark to form the radical ABTS^•+^, and it was used within 2 days. The radical working solution was prepared by diluting the stock solution with PBS (pH 7.4) to an absorbance of 0.70 ± 0.05 at 734 nm. 15 µL of extracts (0.000625–0.01 mg/mL) or compounds (0.0015–0.0075 mM) solutions were mixed with 1485 µL of ABTS^•+^ working solution. The decrease of absorbance was measured at 734 nm by a Thermo Evolution 201 UV–visible spectrophotometer (Thermo Fisher Scientific Italia, Milan, Italy), after 1 min of incubation at room temperature, in reference to a blank (PBS without ABTS^•+^). The scavenging percentage of ABTS^•+^ was calculated relating to Trolox (a water-soluble analog of vitamin E adopted as an antioxidant standard). Antioxidant activity was expressed as mmol Trolox equivalent (TE)/mg extract or mmol compound. A high TEAC value indicated a high level of antioxidant activity.

### 3.8. UHPLC-UV-ESI-HRMS Analysis

UHPLC separation was achieved with a Kinetex C_18_ (100 × 2.1 mm i.d., 2.6 µm) column protected by a C18 Guard Cartridge (2.1 mm i.d.), both from Phenomenex (Torrance, CA, USA) held at 30 °C. The mobile phase consisted of water (A) and MeOH (B), both containing 0.1% HCOOH. The following elution gradient was used: 0–6 min, 5–20% B; 6–10 min, 20–35%, B; 10–15 min, 35–50% B; 15–22 min, 50–70% B; 22–27 min, 70–98% B. After each injection, the column was washed with 100% B for 4 min and re-equilibrated (5 min). A flow rate of 0.4 mL/min and an injection volume of 5 µL were used. Detection by diode array was performed at three wavelengths: 254, 278 and 330 nm and the UV spectra were recorded over a 200–600 nm range. The HRMS and HRMS/MS were performed with an ESI source operating in the ion negative mode. High-purity nitrogen (N_2_) was used as both drying gas and nebulizing gas, and ultra-high pure helium (He) as the collision gas. The operating parameters were optimized as follows: source voltage 3.5 kV, capillary voltage –72 V, tube lens voltage –41.4 V, capillary temperature 280 °C, sheath and auxiliary gas flow (N_2_) 32 e 10 (arbitrary units), respectively. The MS profile was recorded in full scan mode (scan time = 1 micro scans and maximum inject time 500 ms) with resolution of 60,000. For the HRMS/MS acquisitions, a data-dependent method, setting the normalized collision energy in the ion trap of 35%, was used.

### 3.9. Quantitative HPLC Analysis

The HPLC and DAD detection equipment and conditions were the same used for qualitative analysis. The UV chromatograms were recorded at 254 and 278 nm for quantification of ellagic acid (EA) and chestanin, respectively. Calibration external standard method was used to quantify two compounds in CSB extracts (1 and 3 mg mL^–1^). Mixtures of 2 reference standards at different concentrations (six levels in triplicate; EA range 1.5–50 µg/mL; chestanin range 25–400 μg/mL) were used to produce calibration curves. UV peak areas of the external standard (at each concentration) were plotted against the corresponding standard concentrations (µg/mL) using weighed linear regression to generate standard curves. For the linear regression of external standards, R^2^ values were 0.9987 and 0.9993 for EA and chestanin, respectively. The amount of the compounds was finally expressed as micrograms per milligram of extracts. Data are reported in Table 1 as mean standard deviation (SD) of triplicate determinations.

### 3.10. Isolation and Identification of Compounds

A portion of CSB-B (2.0 g) was fractionated over a Sephadex LH-20 column (1 m × 3 cm i.d.) with MeOH as eluent at flow rate 1 mL/min. Fractions of 8 mL each were collected and combined in six major groups (**I**-**VI**) based on their Rf in TLC analysis [(Si-gel, n-BuOH–AcOH–H_2_O (60:15:25)]. Fractions **I**-**V** were purified by RP-HPLC on a Luna C_8_ column (flow rate 2.0 mL/min). Fraction **I** (100.1 mg) was chromatographed with the elution solvent MeOH/H_2_O 6.5:3.5 *v*/*v*, giving compound (**36**) (1.3 mg, *t_R_* = 15 min). Fraction **II** (152.8 mg) was separated using MeOH/H_2_O 5.5:4.5 *v/v* as solvent system to obtain compounds (**29**) (3.3 mg, *t_R_* = 9 min), (**31**) (1.1 mg, *t_R_* = 10 min), and (**28**) (1.8 mg, *t_R_* = 18 min. Fraction **III** (184.1 mg) was purified with MeOH/H_2_O 5:5 *v/v* as solvent system and afforded compounds (**21**) (6.9 mg, *t_R_* =9 min), (**20**) (3.1 mg, *t_R_* = 11 min), (**25**) (3.7 mg, *t_R_* = 12 min), (**30**) (1.2 mg, *t_R_* = 14 min) and (**32**) (1.0 mg, *t_R_* = 16 min). Fraction **IV** (200.4 mg) was separated using as solvent system MeOH/H_2_O 2:8 *v*/*v* giving compounds (**21**) (3.9 mg, *t_R_* = 10 min), (**17**) (3.7 mg, *t_R_* = 14 min), and (**20**) (2.9 mg, *t_R_* = 38 min). Fraction **V** (114.0 mg) was purified with a solvent system MeOH/H_2_O 4:6 *v/v* and gave compounds (**21**) (6.4 mg, *t_R_* = 14 min), and (**33**) (1.8 mg, *t_R_* = 23 min). Finally, fraction **VI** (253.6 mg) was purified by RP-HPLC using MeOH/H_2_O 3.5:6.5 *v*/*v* on a C_18_ Synergy Fusion column, (flow rate 1.8 mL/min) to obtain compounds (**11**) (2.2 mg, *t_R_* = 12 min), (**9**) (11.5 mg, *t_R_* = 14 min), and (**21**) (22.2 mg, *t_R_* = 28 min).

The NMR data of all the isolated compounds corresponded to those reported in literature [30,43,44,45,46,47,48,49,50,51], the ESI-MS data are reported in Table 2.

### 3.11. Antifungal Activity

#### 3.11.1. Fungal Pathogens

The strains of the pathogens *Alternaria alternata*, *Botrytis cinerea*, and *Fusarium solani* used in this work were taken from CREA-Pontecagnano (Salerno, Italy) collection, maintained at 20 °C on PDA slant.

#### 3.11.2. In Vitro Antifungal Assays

The inhibitory effect of CSB-H, CSB-M, and CSB-A extracts on mycelial growth of *A. alternata*, *B. cinerea*, and *F. solani* was assayed using an amended plate technique [52]. Extracts were previously sterilized through 2 h UV exposition (λ = 275 nm). CSB-M was dissolved in DMSO, then poured in sterile 0.1 × PDA, while CSB-H and CSB-A were directly dissolved into sterile 0.1 × PDA until to obtain the final concentrations in the range 30–70 mg/mL to assay *B. cinerea*, and in the range 2.5–50 mg/mL to assess the susceptibility of the remaining two fungi. The final concentration of DMSO in the plates never exceed 2.5%. Not amended plates were used as control. A fungal plug (0.5 cm diameter) taken from the edge of a fresh culture, was transferred onto the center of the plate. Plates were incubated in the dark at 25 °C according to a randomized design. Each treatment was tested in triplicate and the experiment was conducted twice. The mycelial growth diameter was measured when fungus completely covered the control plates. The inhibition percentage of fungal growth was calculated as follows:

Fungal growth inhibition (%) = 100 × [DC − DT/DC]

where DC and DT are the average diameters of fungal colony in the control and in the treated plates, respectively. The EC_50_ values were calculated by linear regression of Probit of the fungal inhibition percentage and the log of the extract concentrations [52]. The EC_50_ was defined as the concentration required to inhibit fungal growth by 50% of the control.

#### 3.11.3. Spore Germination Assay

In order to collect conidia of *A. alternata, F. solani*, and *B. cinerea*, each fungus was transferred onto PDA in Petri dishes (9 cm diameter) and incubated at 25 °C for 7–10 d in darkness. The sporulated plates were then flooded with sterile distilled water and gently rubbed with a sterile bent plastic rod to release conidia. Conidia suspensions were filtered on synthetic filtering wool to remove mycelia fragments, then concentration was determined using a Burker chamber and adjusted to 1 × 10^6^ conidia mL^−1^ by dilution [15]. To assay the effects of chestnut extracts and pure compounds on fungal conidia germination, a 0.1 × PDB microculture method was used. The stock solutions of CSB-M, EA (pure compound), and commercial fungicides (iprodione and carbendazim) were prepared in DMSO. The extracts CSB-H and CSB-A were dissolved directly into PDB, while chestanin (pure compound) was solubilized into water. Aliquots of spore suspension (10 μL, 10^6^ spores/mL) were pipetted in 1.5 mL-tubes containing final concentration 0.1 × PDB supplemented with extract, pure compound or commercial fungicide solutions (final volume 100 μL). Extracts were assayed at concentrations ranging between 10–50 mg/mL, 15–50 mg/mL and 5–30 mg/mL, for *A. alternata*, *B. cinerea*, and *F. solani*, respectively. While pure compounds were used at concentrations included in the range 0.005–1 mg/mL in *A. alternata* experiments and 0.010–2 mg/mL with the other two fungi. The commercial fungicides dissolved into DMSO were used as positive controls and tested at concentration of 0.00025–0.25 mg/mL, while DMSO (2.5%) and not-amended cultures were used as negative controls. Treatments were three-replicated. Tubes were incubated at 25 °C for 24 h according to a complete randomized design. After that, the samples were observed under the light microscope (Leitz, Wetzlar, Germany) (40× magnification) to examine the occurrence of germination. A spore was considered germinated when the length of the germ tube equaled or exceeded the length of the spore. At least 100 spores of each replicate were observed, then the percentage of the spore germination inhibition was calculated as follows:

Spore Germination inhibition (%) = 100 × [% GC_sample_ − %GC_control_/%GC_sample_]

where GC sample and GC control are average percentage of germinated conidia of treatment and control (only vehicle), respectively. The experiment was repeated three times. The EC_50_ values were determined as the extract concentration inhibiting germination at 50% of the untreated control.

### 3.12. Statistical Analysis

The free radical scavenging and total phenol content data were subjected to one-way analysis of variance (ANOVA) followed by Tukey HSD test (*p* ≤ 0.05), using GraphPad Prism version 7.00 for Windows. The Log-Dose-Response curves allowed determination of EC_50_ values for the fungal bioassay according to the Probit analysis. The 95% confidence limits for the range of EC_50_ values were determined and they were considered to be significantly different, if the 95% confidence limits did not overlap. The Chi-square test was performed to compare observed with expected dose-response dataset and the resulting *p* level (>0.05), associated to each EC_50_ value, indicates the goodness of fit between the distributions [52].

## 4. Conclusions

The presented research reports for the first time an accurate profiling of chestnut burs organic and aqueous extracts, by ultra-high-performance liquid chromatography coupled with UV and high-resolution mass spectrometry detectors (UHPLC-UV-HRMS). The comprehensive chemical analysis of the solid waste led to identify several compounds, mainly gallic and ellagic acid derivatives, hydrolysable tannins, and glycosylated flavonols. Extracts and compounds showed a significant antioxidant and plant pathogens inhibitory activity. These results can support the exploitation of natural compounds from renewable sources useful as sustainable alternatives of synthetic fungicides in managing fruit and vegetable diseases.

## Figures and Tables

**Table 1:** Free radical-scavenging activity, total phenolic content (TPC), and amount of Chestanin (**21**) and ellagic acid (EA, **27**) in *C. sativa* burs (CSB) extracts.

	DPPH Test (EC_50_^a^ µg/mL Extract or Phenol) ^b^	TEAC Value ^c^ (mM Trolox/mg Extract or mM Compound) ^b^	TPC ^b^ g GAE ^d^/100 g Extract	Chestanin ^e^ (mg/g)	EA ^e^ (mg/g)
CSB-H	24.94 ± 0.46	3.00 ± 0.22	20.60 ± 0.85	3.21	7.40
CSB-M	12.64 ± 0.12	3.52 ± 0.13	26.42 ± 0.95	13.34	79.32
CSB-A	22.38 ± 2.80	2.71 ± 0.71	20.26 ± 0.14	1.10	5.26
quercetin 3-*O*-β-d-glucopyranoside (**25**)	2.98 ± 0.84	3.39 ± 0.11			
EA (**27**)	2.40 ± 0.24	4.98 ± 0.21			
Chestanin (**21**)	16.62 ± 0.84	1.05 ± 0.14			
gallic acid ^f^	1.23 ± 0.15	3.49 ± 0.21			

^a^ EC_50_ = the concentration (in micrograms per milliliter) of sample necessary to decrease the initial DPPH concentration by 50%; ^b^ Mean ± SD of three determinations; different letters in the same column indicate significantly different (*p* < 0.05); ^c^ TEAC value = concentration of standard trolox with the same antioxidant capacity as 1mg/mL of the tested extract or 1mM of the antioxidant compounds; ^d^ Gallic acid equivalent; ^e^ determined by UHPLC-UV; ^f^ Positive control of the ABTS^•+^ and DPPH^•^ assays.

**Table 2:** UHPLC-HRMS data of compounds detected in CSB extracts.

N ^a^	RT (min)	[M − H]^−^ (*m*/*z*)	Molecular Formula	Error ppm	Diagnostic Product Ions (*m*/*z*) ^b^	Compound	Ref.
**1**	1.97	1101.0672	C_48_H_30_O_31_	−1.4	1057 [M − H − CO_2_]^−^	valoneoyl–NHTP–glucose (vescavaloninic/castavaloninic acid)	[24]
**2**	2.45	933.0613	C_41_H_26_O_26_	−1.6	915 [M − H − H_2_O]^−^, 889 [M − H − CO_2_]^−^, 871 [M − H − H_2_O − CO_2_]^−^, 631 [M − H − EA]^−^, 613 [M − H − EA − H_2_O]^−^, 587 [M − H − EA − CO_2_]^−^, 569 [M − H − EA − H_2_O − CO_2_]^−^	HHDP–NHTP–glucose (castalagin/vescalagin)	[28]
**3**	2.59	783.0663	C_34_H_24_O_22_	−1.6	481 [M − H − EA]^−^, 301 [EA − H]^−^ (C_14_H_5_O_8_ 1.8 ppm)	diHHDP–glucose (pedunculagin)	[28]
**4**	3.16	947.0772	C_42_H_28_O_26_	−1.4	915 [M − H − CH_3_OH]^−^	methyl–HHDP–NHTP–glucose (methylvescalagin)	[24]
**5**	4.01	783.0661	C_34_H_24_O_22_	−1.9	481 [M − H − EA]^−^, 301 [EA − H]^−^ (C_14_H_5_O_8_ 1.8 ppm)	diHHDP-glucose (pedunculagin)	[28]
**6**	4.71	953.0882	C_41_H_30_O_27_	−0.9	909 [M − H − CO_2_]^−^, 785 [M − H − C_7_H_4_O_5_]^−^	galloyl-chebuloyl-HHDP-glucose (chebulagic acid)	[29]
**7**	4.74	613.0454	C_27_H_18_O_17_	−0.9	595 [M − H − H_2_O]^−^, 523 [M − H − C_3_H_6_O_3_]^−^, 493 [M − H − C _4_H_8_O_4_]^−^	castacrenin C	[30]
**8**	5.32	785.0820	C_34_H_26_O_22_	−1.4	633 [M − H − galloyl]^−^, 615 [M − H − GA]^−^, 483 [M − H − EA]^−^, 301 (C_14_H_5_O_8_ 2.1 ppm)	digalloyl-HHDP-glucose (tellimagrandin I)	[31]
**9**	5.58	613.0455	C_27_H_18_O_17_	−0.8	595 [M − H − H_2_O]^−^, 523 [M − H − C_3_H_6_O_3_]^−^, 493 [M − H − C_4_H_8_O_4_]^−^	castacrenin B ^f^	[30]
**10**	5.71	1115.0825	C_49_H_32_O_31_	−1.3	1097 [M − H − H_2_O]^−^, 1071 [M − H − CO_2_]^−^, 1053 [M − H − H_2_O − CO_2_]^−^, 933 [M − H − C_8_H_6_O_5_]^−^, 569 [M − H − C_8_H_6_O_5_ − EA − CO_2_ − H_2_O]^−^	methylvaloneoyl–NHTP–glucose (vescavaloninic/castavaloninic acid methyl ester)	
**11**	5.98	613.0454	C_27_H_18_O_17_	−0.9	523 [M − H − C_3_H_6_O_3_]^−^, 493 [M − H − C_4_H_8_O_4_]^−^	Castacrenin A ^f^	[30]
**12**	6.20	935.0769	C_41_H_28_O_26_	−1.8	917 [M − H − H_2_O]^−^, 873 [M − H − H_2_O − CO_2_]^−^, 783 [M − H − GA]^−^ 633 [M − H − EA]^−^,	galloyl-diHHDP-glucose (stachyurin/casuarinin)	[31]
**13**	6.53	933.0609 ^d^	C_82_H_52_O_52_	1.0	1565 [M − H − EA]^−^, 915 [HHDP-NHTP-glucose − H_2_O]^−^, 633 [galloyl-diHHDP-glucose − EA]^−^, 631 [HHDP-NHTP-glucose − EA]^−^	HHDP-NHTP-glucose-galloyl-diHHDP-glucose (cocciferin d2)	[24]
**14**	6.93	967.1035	C_42_H_32_O_27_	−1.3	785 [M − H − C_8_H_6_O_5_]^−^	galloyl-methylchebuloyl-HHDP-glucose (chebulagic acid methyl ester)	[32]
**15**	7.76	785.0822	C_34_H_26_O_22_	−1.3	633 [M − H − galloyl]^−^, 615 [M − H − GA]^−^, 483 [M − H − EA]^−^, 301 (C_14_H_5_O_8_ 2.2 ppm)	digalloyl-HHDP-glucose (tellimagrandin I)	[31]
**16**	7.97	953.0882	C_41_H_30_O_27_	−0.9	909 [M − H − CO_2_]^−^, 785 [M − H − C_7_H_4_O_5_]^−^	galloyl-chebuloyl-HHDP-glucose (chebulagic acid)	[29]
**17**	8.18	637.1028	C_27_H_26_O_18_	−1.2	467 [M − H − GA]^−^, 305 [M − H − GA − hex]^−^	Chesnatin ^f^	
**18**	8.65	967.1038	C_42_H_32_O_27_	−1.0	785 [M − H − C_8_H_6_O_5_]^−^	galloyl-methylchebuloyl-HHDP-glucose (chebulagic acid methyl ester)	[32]
**19**	9.49	637.1032	C_27_H_26_O_18_	−1.0	593 [M − H − CO_2_]^−^, 469 [M − H − C_7_H_6_O_5_]^−^	isochesnatin ^f^	
**20**	10.35	469.0972	C_20_H_22_O_13_	−1.0	169 [GA − H]^−^ (C_7_H_5_O_5_ 1.8 ppm),	cretanin ^f^	
**21**	11.61	937.1871	C_40_H_42_O_26_	−1.0	637 [M − H − C_13_H_16_O_8_]^−^, 467 [M − H − C_20_H_22_O_13_]^−^	chestanin ^f^	
**22**	12.25	351.1076	C_17_H_20_O_8_	0.5	163 [M − H − C_8_H_12_O_5_]^−^	methyl coumaroyl quinate	
**23**	12.43	615.0977	C_28_H_24_O_16_	−0.5	463 [M − H − galloyl]^−^, 301 [Ag − H]^−^ (C_15_H_9_O_7_ 0.4 ppm)	quercetin-galloyl-hexoside	[28]
**24**	12.64	937.1865	C_40_H_42_O_26_	1.7	467 [M − H − C_20_H_22_O_13_]^−^	chestanin isomer ^f^	
**25**	13.48	463.0867	C_21_H_20_O_12_	−1.5	301 [Ag − H]^−^ (C_15_H_9_O_7_ 1.2 ppm)	quercetin 3-*O*-β-d-glucopyranoside ^f^	
**26**	13.33	477.0660	C_21_H_18_O_13_	−0.8	301 [Ag − H]^−^ (C_15_H_9_O_7_ 0.8 ppm)	quercetin hexuronoside	[33]
**27**	13.62	300.9982	C_14_H_6_O_8_	1.1	-	ellagic acid ^c^	
**28**	14.78	551.1026 ^e^	C_23_H_22_O_13_	−1.0	343 [M − H − Hex]^−^	Ellagic acid 3,3′,4-trimethoxy 4′-*O*-β-d-glucopyranoside ^f^	
**29**	14.91	447.0916	C_21_H_20_O_11_	−1.3	327 [M − H − C_4_H_8_O_4_]^−^, 285 [Ag − H]^−^ (C_15_H_9_O_6_ 1.1 ppm)	Astragalin ^f^	
**30**	14.94	491.0815	C_22_H_20_O_13_	−1.0	315 [Ag − H]^−^ (C_16_H_11_O_7_ 0.6 ppm), 301 [M − H − Hexu−CH_3_]^−^	Isorhamnetin hexuronoside ^f^	
**31**	15.11	477.1024	C_22_H_22_O_12_	−0.6	315 [Ag − H]^−^ (C_16_H_11_O_7_ 1.8 ppm)	isorhamnetin 3-*O*-β-d-glucopyranoside ^f^	
**32**	15.29	623.1599	C_28_H_32_O_16_	−1.2	315 [Ag − H]^−^ (C_16_H_11_O_7_ 1.6 ppm)	isorhamnetin-rhamnoside-hexoside	[28]
**33**	16.8	609.1231	C_30_H_26_O_14_	−1.3	463 [M − H − coumaroyl]^−^, 301 [Ag − H]^−^ (C_15_H_9_O_7_ 0.8 ppm)]	quercetin 3-*O*-(6″-*O*-trans-*p*-coumaroyl)-β-d-glucopyranoside ^f^	
**34**	17.84	593.128	C_30_H_26_O_13_	−1.5	447 [M − H − coumaroyl]^−^, 285 [Ag − H]^−^ (C_15_H_10_O_6_ 1.2 ppm)	kaempherol coumaroyl hexoside	[28]
**35**	18.00	593.12748	C_30_H_26_O_13_	−2.5	447 [M − H − coumaroyl]^−^, 285 [Ag − H]^−^ (C_15_H_10_O_6_ 1.7 ppm)	Tiliroside ^c^	
**36**	18.04	535.1076 ^e^	C_23_H_22_O_12_	−1.1	343 [M−H dHex]^−^	Ellagic acid 3,3′,4-trimethoxy 4′-*O*-α-l-rhamnopyranoside ^f^	
**37**	18.22	623.1388	C_31_H_28_O_14_	−1.1	477 [M − H − coumaroyl]^−^, 315 [Ag − H]^−^ (C_16_H_12_O_7_ 1.5 ppm)	isorhamnetin coumaroyl hexoside	[28]
**38**	19.16	593.1284	C_30_H_26_O_13_	−1.0	285 [Ag − H]^−^ (C_15_H_10_O_6_ 1.5 ppm)	kaempherol coumaroyl hexoside	[28]
**39**	19.74	635.1282	C_32_H_28_O_14_	−2.1	575 [M − H − acetyl]^−^, 285 [Ag − H]^−^ (C_15_H_10_O_6_ 2.7 ppm)	kaempherol acetyl coumaroyl hexoside	[28]
**40**	21.09	739.1648	C_39_H_32_O_15_	−1.3	593 [M − H − coumaroyl]^−^, 453 [M − H − Kaempferol]^−^, 285 [Ag − H]^−^ (C_15_H_10_O_6_ 2.4 ppm)	kaempferol dicoumaroyl hexoside	[28]
**41**	21.9	781.1753	C_41_H_34_O_16_	−1.3	635 [M − H − coumaroyl]^−^, 495 [M − H − Kaempferol]^−^, 285 [Ag − H]^−^ (C_15_H_10_O_6_ 2.2 ppm)	kaempherol acetyl dicoumaroyl hexoside	[28]
**42**	22.11	781.1747	C_41_H_34_O_16_	−2.0	635 [M − H − coumaroyl]^−^, 495 [M − H−Kaempferol]^−^, 285 [Ag − H]^−^ (C_15_H_10_O_6_ 2.2 ppm)	kaempherol acetyl dicoumaroyl hexoside	[28]

Abbreviations: GA: gallic acid; EA: ellagic acid; dHex: loss of deoxyhexose (−146 Da); Hex: loss of hexose (−162 Da); Hexu: loss of hexuronose (−176 Da); Ag: aglycone. ^a^ Compounds are numbered according to their elution order; ^b^ In bold the base peak of MS/MS spectrum; ^c^ Compared with reference standards; ^d^ *m*/*z* values corresponding to [M − 2H]^−2^; ^e^ *m*/*z* values corresponding to [M + HCOOH − H]^−^; ^f^ The identification of these compounds was corroborated by isolation procedure and NMR spectra analys.

**Table 3:** EC_50_ of CSB extracts inhibiting *Alternaria alternata*, *Botrytis cinerea* and *Fusarium solani* mycelial growth.

	EC_50_ Growth Inhibition (mg mL^−1^)	95% Fiducial Limits		Chi-square Test (*p* Value) ^a^
		Lower	Upper	
*Alternaria alternata*				
CSB-H	8.71	7.16	10.26	1.00
CSB-M	6.29	5.71	6.87	0.88
CSB-A	14.53	13.59	18.17	0.99
*Botrytis cinerea*				
CSB-H	>70			
CSB-M	64.98	61.85	68.11	0.88
CSB-A	>70			
*Fusarium solani*				
CSB-H	14.13	11.35	16.91	0.55
CSB-M	6.04	5.22	6.85	0.99
CSB-A	15.51	11.19	19.83	0.93

*^a^* Chi-square value, significant at *p* < 0.05 level.

**Table 4:** EC_50_ of CSB inhibiting *Alternaria alternata*, *Botrytis cinerea* and *Fusarium solani* spore germination.

	EC_50_ Germination Inhibition (mg mL ^−1^)	95% Fiducial Limits		Chi-square Test (*p* Value) ^a^
		Lower	Upper	
*Alternaria alternata*				
CSB-H	11.17	8.91	27.77	0.53
CSB-M	2.66	1.48	8.70	1.00
CSB-A	5.48	1.14	9.82	0.24
*Botrytis cinerea*				
CSB-H	>50			
CSB-M	16.33	4.85	27.81	0.61
CSB-A	>50			
*Fusarium solani*				
CSB-H	10.52	5.28	15.76	0.72
CSB-M	2.22	1.84	2.60	0.95
CSB-A	6.80	5.18	8.42	0.99

*^a^* Chi-square value, significant at *p* < 0.05 level.

**Table 5:** EC_50_ of pure compounds compared to synthetic fungicides (iprodione, and carbendazim) inhibiting *Alternaria alternata*, *Botrytis cinerea* and *Fusarium solani* spore germination.

	EC_50_ Growth Inhibition (µg mL^−1^)	95% Fiducial Limits		Chi-square Test (*p* value) ^a^
		Lower	Upper	
*Alternaria alternata*				
EA (**27**)	13.33	12.77	13.90	0.99
Chestanin (**21**)	561.56	544.57	578.54	0.92
Iprodione	0.85	0.70	0.99	1.00
*Botrytis cinerea*				
EA (**27**)	112.64	8.89	219.11	1.00
Chestanin (**21**)	>2000			
Iprodione	37.36	18.90	58.10	0.99
*Fusarium solani*				
EA (**27**)	21.27	15.57	26.43	1.00
Chestanin (**21**)	>2000			
Carbendazim	14.29	6.03	21.97	0.99

*^a^* Chi-square value, significant at *p* < 0.05 level.
